# The Case-History of Hæmorrhages in the Early Months of Pregnancy

**Published:** 1908-02-29

**Authors:** 


### Gynæcology.

### THE CASE-HISTORY OF HÆMORRHAGES IN THE EARLY MONTHS OF PREGNANCY.

There is, perhaps, no single common disorder in the diagnosis of which a detailed history is of greater value than in early abortions. Not only so, there can be very few in which the unsifted statements of the patient, though made in perfectly good faith, are so misleading. Now this condition is so common that a majority of the married women in the population have probably experienced it at least once, and its importance and its effects, therefore, exceed in magnitude those of all other causal agents of uterine hæmorrhage in early pregnancy.

In taking the history of any case of bleeding from the genital tract in a woman of the child-bearing age, at the outset an attempt must be made to establish the date of the last normal period; normal, that is to say, for the patient under consideration. Unless special stress is laid on the details of this inquiry confusion is almost certain to arise; for most women will regard and speak of as menstruation any hæmorrhage, unless very great, whether it coincides with the expected onset of that condition or not. Having settled this, the history of the subsequent departures from the normal is to be extracted *seriatim*, the utmost care being taken to confine the narrator to observed facts, and to exclude her deductions.

The manner in which this history should be taken it is not necessary to consider here very minutely; the existing text-books on gynæcology deal amply with the subject. But it may be noted that the one point which is most suggestive to the physician frequently seems of quite minor importance to the patient; that is, the missing of one (or more) periods without any reasonable explanation. When a married woman fails without warning to menstruate, and then has an unusually copious hæmorrhage two or three weeks later, it is surprising that it should seldom enter her head that the most likely explanation of this is the termination of an early gestation. This, however, is a statement of truth which the medical man should never fail to bear in mind, and the assertion that a period has been so many days or weeks late calls for a most careful review of all previous and subsequent symptoms, and a no less careful investigation of the physical signs.

The cross-examination includes questions as to the passage of clots, formed products, membrane, and so forth; but in practice there is not often anything worthy of notice disclosed. It is, in fact, unreasonable to expect the laity to distinguish blood clot, placental tissue, endometrium, and mucus—and, in fact, the patients' answers to these inquiries seldom yield any reliable information.

When the complete account of the present illness has been obtained it is essential to inquire carefully into the past menstrual and puerperal history, and into the general health. A gradually diminishing loss may signify chlorosis or some graver form of anæmia, tuberculosis, diabetes, or many other debilitating constitutional conditions, whether associated, or more probably not, with pregnancy. Frequent miscarriages should suggest questions about previous peri-uterine inflammation, syphilis, fibroid, or any of the local and general causes of repeated abortion. The consideration of previous labours and their consequences may be enough to establish a strong probability of endometritis, which is believed to be a fertile source of early abortion.

Family history is much less relevant. In a patient over, say, 35 with a suspicious story of pain and discharge with hæmorrhage, it might be worth while to ask for the family experience of malignant disease: this, however, would come just as well after the physical examination, if the latter seemed to call for it; for needless anxiety and morbid fancies are fatally easy to arouse in a patient with any form of pelvic abnormality. Hæmophilia, too, is described in text-books as an occasional cause of early abortion: this disease is well-known to be extremely rare in women, and the reason is readily guessed when one thinks for a moment of the phenomena of gestation, whether carried to term or ended prematurely. In either case, if women suffered equally with men from this diathesis, the breed of hereditary sufferers would be completely extinct in a few years. For practical purposes we can afford to regard hæmophilia as almost negligible in the history of an abortion.

It is, of course, impossible to decide from the symptoms alone whether an abortion is threatened or actual; and if the latter, whether complete or incomplete. Indeed, it is in many cases impossible to be certain that a pregnancy has existed at all. Physical examination can alone decide this. Beyond that, even when gestation has been proved, there are other causes of uterine hæmorrhage besides abortion. Thus, in the diagnosis of ectopic gestation before rupture of the sac, the history is all-important: for the physical signs presented are as a rule just as compatible with a small ovarian or mesometric cyst, or a pedunculated myoma. That rare condition, hydatiform mole, is not commonly seen in quite the early months of pregnancy; but, although it is seldom diagnosed before the discharge of the typical products from which the condition is named, it is by no means rare for the clinician to recognise when that occurs what it was that the combined history and signs had been indicating. The failure to diagnose hydatiform mole is generally due to an overriding of the significance of symptoms by that of apparently contradictory signs: a history very carefully taken in writing is the best safeguard against this.

Tubal mole, tubal abortion, rupture of a tubal gestation are other conditions in which a diagnosis can sometimes be hazarded almost from the history alone, though equally they often give rise to symptoms which may appear obscure. The lesson to be driven home is this, that second as a means of diagnosis as history must always stand to pelvic examination, no pains to obtain it correctly and clearly at the outset should be shirked: no time thus spent is likely to be wasted, and many errors which may entail disastrous consequences will be avoided.

